# Protective Legislation for Dentistry

**Published:** 1881-02

**Authors:** 


*Protective Legislation for Dentistry* is commonly misunderstood, it would seem, from the action of the Committee in the U. S. Senate, to whom was referred the bill to regulate the Practice of Dentistry in the District of Columbia. The conference had by those who were mainly instrumental in preparing the bill with the members of the Senate, shows plainly that our representatives in Congress assembled had not the slightest idea of the situation.

One Senator gravely said that if he chose to patronize a blacksmith for the operation of tooth extraction, he could not see why he should not do so, nor could he see why legislation should hinder him. His whole idea seemed to be that "tooth pulling" was the beginning and end of dentistry. And further the idea seemed to prevail that a privileged few wished legislation to enable them to monopolize a trade or business. That the masses themselves needed protection from the ignorant quacks who maltreat those whose misfortunes cause them to fall into their hands, seemed not to have entered the minds of those who were asked for protective laws. If some one could furnish this Committee of Congress with a primer of dental literature, it is possible good might be done. As it is, it is hard to see how any adequate legislation can be had. States seem to be able to grasp the idea that ours is a profession, but the Congress of the United States evidently needs illumination.

